# Effects of interleaved and blocked study on delayed test of category learning generalization

**DOI:** 10.3389/fpsyg.2014.00936

**Published:** 2014-08-22

**Authors:** Paulo F. Carvalho, Robert L. Goldstone

**Affiliations:** Department of Psychological and Brain Sciences, Indiana UniversityBloomington, IN, USA

**Keywords:** category learning, interleaving, blocking, spacing, comparison

## Abstract

Studying different concepts by frequently alternating between them (i.e., interleaving), improves discriminative contrast between different categories, while studying each concept in separate blocks emphasizes the similarities within each category. Interleaved study has been shown to improve learning of high similarity categories by increasing between-category comparison, while blocked study improves learning of low similarity categories by increasing within-category comparison. In addition, interleaved study presents greater temporal spacing between repetitions of each category compared to blocked study, which might present long-term memory benefits. In this study we asked if the benefits of temporal spacing would interact with the benefits of sequencing for making comparisons when testing was delayed, particularly for low similarity categories. Blocked study might be predicted to promote noticing similarities across members of the same category and result in short-term benefits. However, the increase in temporal delay between repetitions inherent to interleaved study might benefit both types of categories when tested after a longer retention interval. Participants studied categories either interleaved or blocked and were tested immediately and 24 h after study. We found an interaction between schedule of study and the type of category studied, which is consistent with the differential emphasis promoted by each sequential schedule. However, increasing the retention interval did not modulate this interaction or resulted in improved performance for interleaved study. Overall, this indicates that the benefit of interleaving is not primarily due to temporal spacing during study, but rather due to the cross-category comparisons that interleaving facilitates. We discuss the benefits of temporal spacing of repetitions in the context of sequential study and how it can be integrated with the attentional bias hypothesis proposed by Carvalho and Goldstone (2014a).

## INTRODUCTION

Much of our knowledge is acquired inductively. By studying several examples of a given concept we are able to extract the relevant information from those examples and generalize the concept they instantiate. For example, upon seeing several instances of typical birds one might infer that all birds have beaks and feathers. In the context of inductive learning, the way information is organized can have a deep impact on what is learned. If learning is not equally efficient under different conditions, even though the same information is presented, it becomes particularly relevant to identify not only how different conditions affect learning but also how learning can be optimized (Atkinson, 1972; Pavlik and Anderson, 2008).

Given the potentially large influence of example sequencing, most category learning studies employ a neutral, randomly ordered presentation of exemplars and categories when inductively teaching categories. However, outside the lab, information is not usually sequenced randomly. For example, a typical textbook for “Introduction to Statistics” will start with coverage of descriptive statistics, followed by probability theory and then hypothesis testing, i.e., concepts are blocked. An alternative to the blocked study sequence described above is interleaving different concepts. In interleaved study, different concepts are successively alternated. Put concretely, two possible ways to learn the concepts A, B, and C from examples is by blocking the examples of each concept (e.g., A_1_ A_2_ A_3_ B_1_ B_2_ B_3_ C_1_ C_2_ C_3_), or by interleaving examples of all the concepts (e.g., A_1_ B_1_ C_1_ A_2_ B_2_ C_2_ A_3_ B_3_ C_3_). Importantly, these two schedules of presentation provide different study experiences, which has the potential to change what we learn (e.g., Goldstone, 1996; Schyns and Rodet, 1997), and how well we learn it (e.g., Kornell and Bjork, 2008; Wahlheim et al., 2011).

Research in skill acquisition has demonstrated a clear advantage for interleaved study. For example, Shea and Morgan (1979) had participants learn three different sequences of complex movements in an apparatus where each sequence was prompted by the presentation of a different light color. All participants practiced each sequence 18 times. Critically, for half of the participants the practice of each of the three different sequences was interleaved while for the other half it was blocked by light color. The results showed that during study participants in the blocked condition performed better than those in the interleaved condition. However, this pattern was reversed in a delayed transfer task. These results have been extended to other types of learning, namely concept learning using artist styles (Kornell and Bjork, 2008; Kornell et al., 2010; Kang and Pashler, 2012; Zulkiply and Burt, 2013), butterfly and bird species (Wahlheim et al., 2011; Birnbaum et al., 2013; Zulkiply and Burt, 2013), mathematical and clinical concepts (Rohrer and Taylor, 2007; Taylor and Rohrer, 2010; Zulkiply et al., 2012) as well as novel categories (Zulkiply and Burt, 2013; Carvalho and Goldstone, 2014a,b).

Although a diverse set of concepts has been used to show a learning advantage for interleaved study, a common characteristic is that the items from the to-be-learned categories have a high degree of similarity and are, therefore, hard to discriminate or encode individually (Zulkiply and Burt, 2013; Carvalho and Goldstone, 2014a). Hence, presenting items from different categories close in sequence optimizes discriminative contrast leading to better learning (Kang and Pashler, 2012; Birnbaum et al., 2013; Zulkiply and Burt, 2013; Carvalho and Goldstone, 2014a). Conversely, when each item is significantly different from items in the same and different categories, i.e., when low similarity categories are used, research has shown that blocked study results in improved learning (Kurtz and Hovland, 1956; Whitman and Garner, 1963; Goldstone, 1996; Carpenter and Mueller, 2013; Zulkiply and Burt, 2013; Carvalho and Goldstone, 2014a). In the case of learning low similarity categories, the difficulty is not primarily in discriminating items from different categories but rather finding similarities within the categories, which is optimized by often repeating the same category close in time (Carvalho and Goldstone, 2014a,b).

It is therefore possible that category learning depends upon the match between the study sequence and the type of category being studied (Zulkiply and Burt, 2013; Carvalho and Goldstone, 2014a) or the learning situation (Carvalho and Goldstone, 2014b; Rawson et al., 2014). However, blocked and interleaved study do not differ only on the type of contrast they emphasize. They also differ in the amount of temporal spacing between successive repetitions of the same category. Interleaving study maximizes the temporal spacing between repetitions, while blocking study minimizes the temporal spacing between repetitions.

Increasing the temporal spacing between verbatim repetitions during study confers significant mnemonic benefits (Glenberg, 1976; Glenberg and Lehmann, 1980; Pashler et al., 2007; Cepeda et al., 2009; Delaney et al., 2010) and it has been proposed that interleaved study benefits for learning are at least in part due to the temporal spacing between repetitions of the same category (Shea and Morgan, 1979; Lee and Magill, 1985). Interleaved study does not involve temporally spaced token repetitions but rather temporally spaced repetitions of the same category type. When study is blocked by category, even though different specific items might be presented on successive study trials, the same category response is activated for all of them. On the contrary, interleaved study of several categories requires alternating category assignments more frequently. An increased temporal spacing between repetitions of a category increases forgetting of the previous encounter with that category and increases the effort in recalling previous encounters (Bjork and Allen, 1970; Cuddy and Jacoby, 1982; Krug et al., 1990). The increased effort to recall the previous encounter typically results in better long-term retention of the repeated elements across different items of the same category because they were present in both encounters (Vlach et al., 2008, 2012, 2014).

In the case of verbatim repetitions of items, the benefits of spacing are sometimes not seen when the test takes place shortly after learning but are seen at longer retention intervals between study and test (e.g., Peterson et al., 1962a,b; Glenberg and Lehmann, 1980; Bloom and Shuell, 1981; Krug et al., 1990; Rohrer and Taylor, 2006). Thus, the optimal temporal spacing between repetitions depends on the interval between the last study repetition and test (i.e., the retention interval). Initial proposals suggested that increasing the temporal spacing between repetitions improves memory if the retention interval is long (Crowder, 1976) or proportionally longer than the temporal spacing between repetitions during study (Murray, 1983). Recent reviews of the literature indicate that the benefits of increasing the temporal spacing during study depend on the length of the retention interval (Donovan and Radosevich, 1999; Janiszewski et al., 2003; Cepeda et al., 2006). Cepeda et al. (2008) compared a set of temporal lags during study in the context of different retention intervals and noted that when retention interval increases the optimal temporal spacing during study increases as well. Similar evidence of an effect of retention interval length exists in the case of non-verbatim repetitions (Ste-Marie et al., 2004).

While much research has addressed how the benefits of temporally spacing repetitions in word list or paired associates learning tasks, little research has questioned how retention interval and spacing interact in category learning. An important question, therefore, is whether the previously found benefits of blocked study for low similarity categories are only evident at short retention intervals while interleaved study promotes long-term retention. One possible conceptualization of why this might be the case is as follows (see **Table 1** for an overview of these predictions).

**Table 1:** Predictions for each study schedule and category structure for test at different retention intervals.

Study schedule	Attentional bias^a^	Type of category	Test outcome without delay^a^	Test outcome with delay
Blocked	Within-category similarities	Low similarity	Improved learning	Worse learning
		High similarity	Worse learning	Worse learning
Interleaved	Between-category differences	Low similarity	Worse learning	Improved learning
		High similarity	Improved learning	Improved learning

*The kind of study schedule, attentional bias and categories to be learned are described in the first three columns while the last two columns present predictions for learning and retention performance.*

*^a^Predictions based on previous work showing an interaction between category structure and schedule of study (Zulkiply and Burt, 2013; Carvalho and Goldstone, 2014a).*

When discriminating individual items is easy (as in the case of low similarity categories) and the test is immediate, learners might be able to memorize individual items during study and use that memory to categorize novel items during an immediate test (Ashby and O’Brien, 2005). This strategy might provide immediate benefits, similar to what is seen in verbatim massed repetitions of items, but provide a transient memory trace that will result in decreased long-term memory (Glenberg, 1976; Glenberg and Lehmann, 1980). Under this conceptualization, spacing repetitions of low similarity categories (i.e., interleaved study) will result in improved long-term retention of each individual item by increasing the temporal spacing between repetitions. With increasing temporal delays learners might engage in a recursive recollection process (Murray, 1983; Ross, 1984; Ross and Kennedy, 1990; Benjamin and Tullis, 2010; Wahlheim et al., 2014). Every time an item of a category is presented, learners will try to remember the previous items from the same category seen and this recursive retrieval is likely to result in learning benefits (Vlach et al., 2008, 2012, 2014; Birnbaum et al., 2013). When discriminating items is hard, as in the case of high similarity categories, memorizing individual items is less likely and learners will resort to encoding only the relevant features of each category by contrasting them. Interleaved study of categories optimizes attending and encoding these features (Kang and Pashler, 2012; Birnbaum et al., 2013; Carvalho and Goldstone, 2014a). However, increasing the temporal spacing between each category, by, for example, including another task between interleaved presentations of different categories, hinders noticing these differences (Kang and Pashler, 2012; Birnbaum et al., 2013). We will return to the differences between exemplar and rule encoding and its potential importance for understanding sequencing effects in the Section “General Discussion.”

In this paper we investigate the relative benefits of category comparison and temporal delay during study and its interaction with retention interval. We approach this questions by teaching learners two different types of categories: high similarity categories in which all the stimuli are very similar to each other (both within and between the three categories to be learned), and low similarity categories in which any pair of stimuli share relatively few similarities. Additionally, learners’ categorization ability for the items studied and new transfer items was tested both immediately and 24 h after the initial study. To foreshadow, the differential attentional biases promoted by each schedule (interleaving and blocking) will confer differential relative benefits to different types of categories. Moreover, the benefit of the temporal delay between repetitions during study will benefit category learning following interleaved study at increased retention intervals for both category structures.

## EXPERIMENT 1A

### METHOD

#### Participants

**FIGURE 1:**
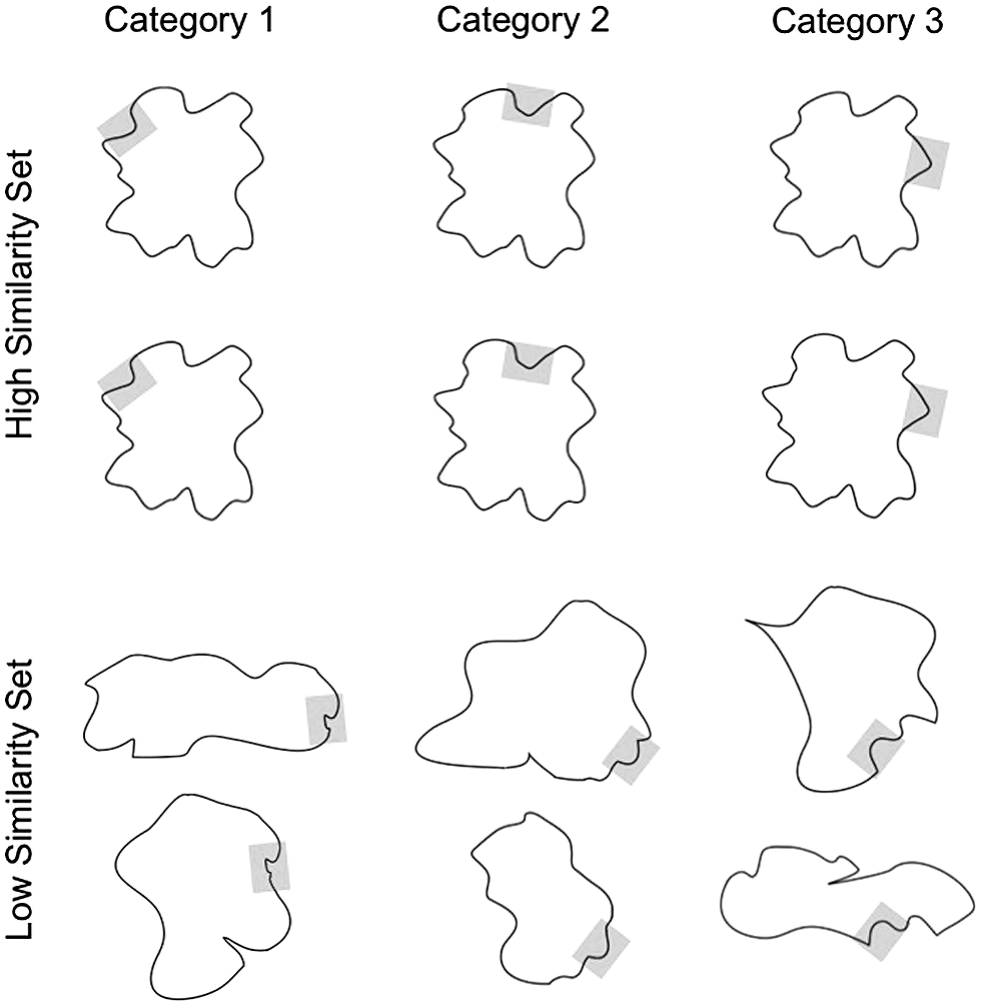
**Examples of stimuli used in the experiments presented here.** All stimuli were created by randomly generating curvilinear segments that were then added together. Each blob was constituted by eight features (each feature was a specific spatial position in the blob).

A total of 178 undergraduate students at Indiana University volunteered to participate in this study in return for partial course credit. Participants were randomly assigned to either the high similarity (*N* = 94) or low similarity (*N* = 84) condition. Data from a total of 65 participants were excluded from analyses due to failure to complete the second session (*N* = 16 for the high similarity condition and *N* = 18 for the low similarity condition), computer error (*N* = 3 for the high similarity condition and *N* = 1 for the low similarity condition), or failure to reach the criterion of 34% correct responses across the four blocks of the initial study phase (*N* = 24 for the high similarity condition and *N* = 3 for the low similarity condition). The higher rate of failure to reach criterion for the high compared to low similarity condition replicates previous studies (Carvalho and Goldstone, 2014a), and is intuitively plausible from an inspection of **Figure 1** and the highly confusable nature of the high similarity stimuli.

#### Apparatus and stimuli

The stimuli used were blob figures (see **Figure 1**). These stimuli were previously used by Carvalho and Goldstone (2014a). All blobs were created by randomly generating curvilinear segments. A single curvilinear segment defined each category and was present in all exemplars of that category. Across all of our experiments, two sets of six categories were used (three categories studied blocked and three studied interleaved, randomly selected for each participant), a low-similarity set and a high-similarity set, for a total of 12 categories. Each category was composed of 16 exemplars.

In the high-similarity set, exemplars shared most of their features with all of the other exemplars in the same category and in each of the other five categories. Moreover, variation within each category was exactly the same for all categories, so that a difference that could exist between two exemplars in category 1 would also exist between two exemplars of each of the other categories in the set. In the low-similarity set, exemplars within each category shared only the category-relevant feature. Moreover, exemplars from different categories differed in all of their features. Some of the exemplars had an overall round shape, and others an overall oblique shape (this variability was equally distributed across categories).

As a cover story, participants were told that a recent expedition to Mars had recovered several cells of alien organisms. Each cell could be categorized into one of three species solely on the basis of its perceptual features. Stimuli were presented on a computer screen, and participants responded by pressing one of three buttons drawn on the screen, with an inconsistent mapping between location of the button and category label.

Each category was composed of a total of 16 blobs. For each subject, eight blobs were randomly selected to be used during study while the remainder were used during test only. Each category was given a novel name, randomly selected for each participant from the following pool: “beme,” “kipe,” “vune,” “coge,” “zade,” and “tyfe” (Hendrickson et al., 2012).

#### Design and procedure

This experiment had four conditions manipulated within-participants (schedule of study: interleaved vs. blocked study; and time of test: immediate vs. 24-h delayed) and two conditions manipulated between-participants (type of category: high similarity vs. low similarity categories). Participants started by completing one of the study conditions and the corresponding immediate transfer test and then completed the next study condition and the immediate test for that condition during their initial visit to the lab (order of conditions was counterbalanced across participants). Participants returned to the lab approximately 24 h after finishing the second transfer test for a follow-up session.

#### Study phase

Each study phase was composed of four blocks of 48 trials each. Each trial started with a presentation of one stimulus in the center of the screen for 500 ms. After the blob was removed, the participant was asked to classify the blob they had just seen into one of three species by clicking the button on the screen with the correct species name. The label of each of the buttons was randomized on each trial so that the absolute position of a button on the screen could not be reliably associated with a category. Immediately after a response was recorded, the blob was presented again in the center of the screen along with the correct category assignment and an indication as to whether the participant’s response was correct or incorrect. Feedback was presented for 2000 ms. A 1000 ms intertrial interval followed and then a new trial began.

The two schedules of study (blocked vs. interleaved) differed only in the frequency of category change during study and the category labels. In the blocked condition, the presented categories alternated 25% of the time, whereas in the interleaved condition, they alternated 75% of the time. Thus, in the interleaved condition, the probability of a blob being followed by a blob of the same category was low, whereas for the blocked condition, this probability was high. We used this probabilistic approach rather than creating purely interleaved or blocked conditions in order to diminish the possibility that participants noticed the pattern of alternation in responses, which would affect categorization accuracy (see Carvalho and Goldstone, 2014a for analysis and discussion of these effects).

#### Immediate transfer test

Immediately after each study phase participants completed a transfer task. This task was composed of a total of 48 trials. Half of the trials were old trials in which an exemplar that had been presented before was presented and the other half were new trials in which a novel exemplar was presented. The new stimuli were similar to the ones studied, with new instantiations of the unique features (i.e., the unique feature presented with different non-diagnostic features). A random sequence of categories was used, meaning that the probability of successive items belonging to the same category was 33%. On each transfer trial the stimulus was presented in the center of the screen for 500 ms. Once the stimulus was removed from the screen, the participants had to categorize it into one of the species they had just studied by clicking one of the buttons on the screen. The label of each of the buttons was randomized on each trial. No feedback was provided during the immediate transfer test.

#### Delayed transfer test

On their second visit to the lab, participants started by completing a refresher training task. The refresher task was given because pilot results indicated that some participants had memory of the previous day’s categorization task, but did not remember which label had been associated with each stimulus type^1^. This refresher task was composed of 24 training trials similar to the study task trials from the previous day, using the same study schedule as in in the previous session. Immediately after the refresher training task, participants completed a transfer test similar to the immediate transfer set they had completed the day before, using the same set of stimuli and with no feedback provided.

### RESULTS AND DISCUSSION

**FIGURE 2:**
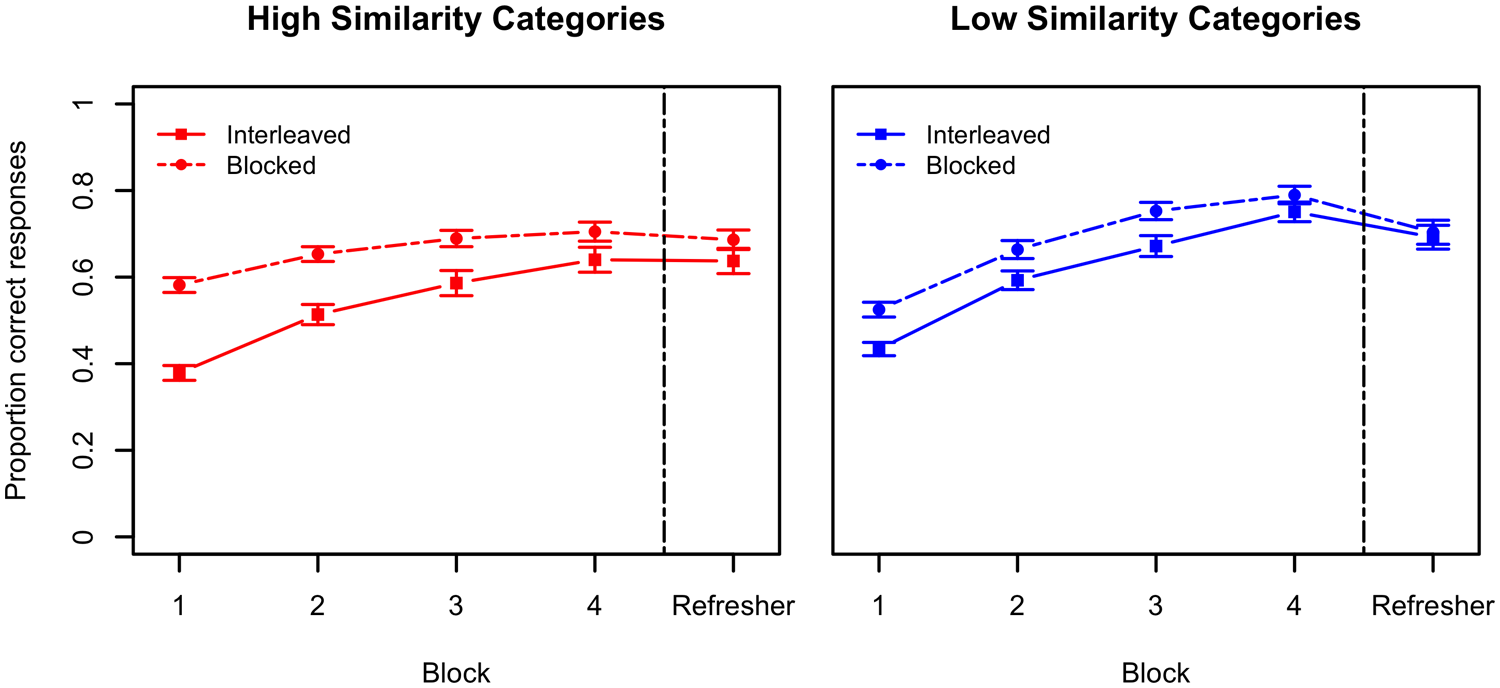
**Performance during the study session and the refresher component for Experiment 1A.** The left panel shows performance for interleaved and blocked study of high similarity categories. The right panel shows performance for interleaved and blocked study for low similarity categories. Error bars indicate standard errors of the means. Chance-level performance in this task was 0.33. The vertical dashed line represents session break.

We begin by analyzing the data from the study phase. These results are depicted in **Figure 2**. First, we focus on performance over the four blocks of the initial study session. One initial question is whether there is an interaction between the type of category and study schedule. This interaction was not reliable (*p* > 0.05), thus study performance seems to be approximately equivalent for each study schedule across the two category structures.

However, performance is overall better during blocked study when compared to interleaved study, *F*(1,111) = 41.51, *p* < 0.0001, $\eta_{G}^{2}$ = 0.09. This result parallels previous evidence (e.g., Shea and Morgan, 1979; Carvalho and Goldstone, 2014a) showing a benefit of blocked study during study. However, this study advantage does not always transfer to an equivalent advantage of blocked study during test. Blocked study presents a higher level of response predictability – this fact might help explain why performance is better during blocked presentation. Finally, low similarity categories are also easier to learn than high similarity ones, resulting in overall better performance, *F*(1,111) = 6.83, *p* = 0.01, $\eta_{G}^{2}$ = 0.03.

Notwithstanding these differences, we see an improvement in the ability to categorize the blobs across the study phase for all conditions, *F*(3,333) = 249.12, *p* < 0.0001, $\eta_{G}^{2}$ = 0.25. However, this improvement is greater for low similarity categories compared to high similarity categories, *F*(3,333) = 10.16, *p* < 0.0001, $\eta_{G}^{2}$ = 0.01 and for interleaved study compared to blocked study, *F*(3,333) = 10.30, *p* < 0.0001, $\eta_{G}^{2}$ = 0.01. These results are also similar to previous evidence comparing interleaved and blocked study.

Finally, we compared performance in the last block of study in day 1 with performance on the refresher of day 2. Overall performance was lower on the second day refresher than on the last block of the study session in day 1, *t*(112) = 3.58, *p* < 0.001. This effect seems to be mostly driven by the results in the low similarity category structure (see **Figure 2**). This slight decrease in performance is expected given the time interval between the last study block and the refresher. No effects of schedule of study, similarity structure of the categories or interaction between the two variables were found for the refresher session (all *p*s > 0.05).

**FIGURE 3:**
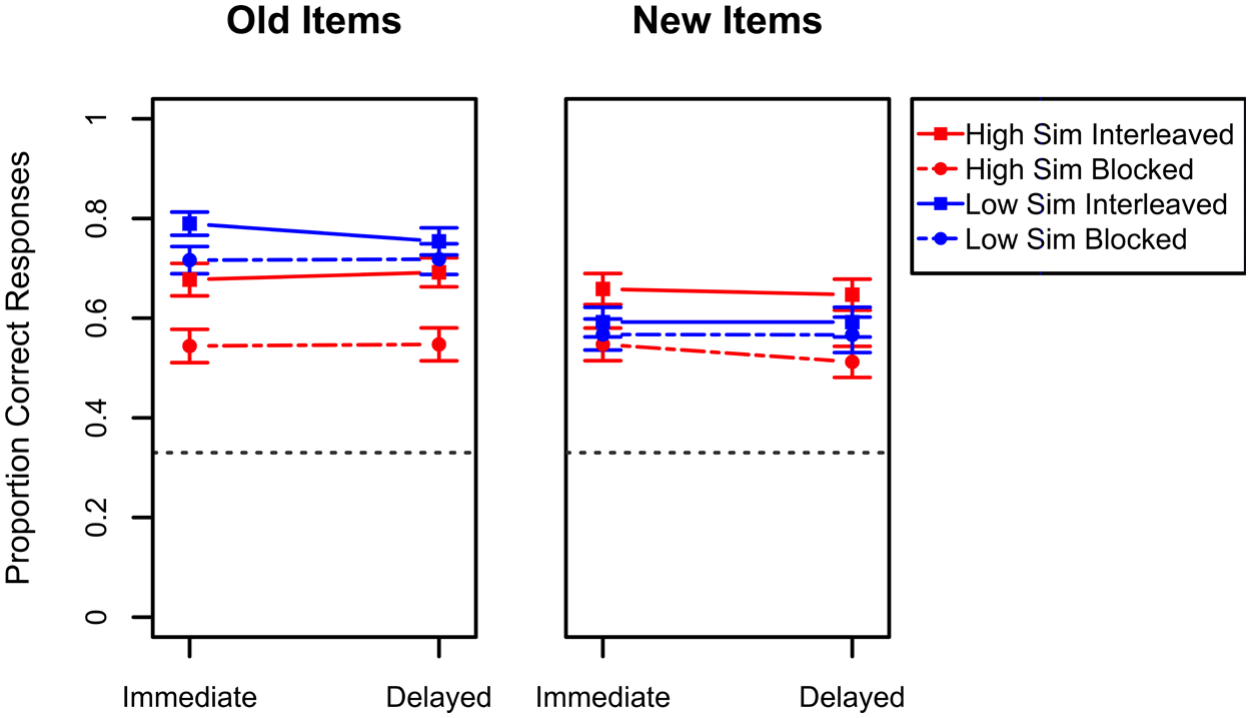
**Performance in the transfer tests of Experiment 1A.** The left panel depicts results for items studied during the study phase while the right panel presents results for items not seen during the study phase. Results for the high similarity categories are presented in red while results for the low similarity categories are presented in blue. For each of these, the dashed lines represent blocked study while the solid lines represent interleaved study. Error bars indicate standard errors of the means. Chance-level performance in this task was 0.33 and is represented in the graphs by the black dashed line.

We now turn our attention to the results during test for both novel and studied items. The main results are depicted in **Figure 3**. As a reminder, there were two test sessions: one that took place immediately after the corresponding study session and another that took place 24 h later. Analyses of these data, revealed a main effect of study schedule, with overall better performance for interleaved study than blocked study, *F*(1,111) = 14.99, *p* < 0.001, $\eta_{G}^{2}$ = 0.04. However, this effect is qualified by a series of relevant interactions.

The three questions of interest relative to performance in the test phase are (1) whether there is an interaction between type of category used and the study schedule used, (2) whether there is an overall improvement in performance following interleaved study between immediate test and the 24 h delayed test, and (3) whether the interaction pattern seen in immediate transfer tests changes when transfer is tested 24 h later. As can be seen from the **Figure 3**, there is an interaction between the type of category used and the schedule of study, *F*(1,111) = 4.26, *p* = 0.04, $\eta_{G}^{2}$ = 0.009. This interaction shows that while interleaved study results in the best transfer performance for high similarity categories, this advantage is considerably reduced for low similarity categories. In the case of low similarity categories, no schedule of presentation seems to result in overall better performance. Moreover, this interaction does not change with transfer test time, i.e., it remains the same 24 h after study. However, a statistically reliable three-way interaction between category type, schedule of study and test session, *F*(1,111) = 5.32, *p* = 0.02, $\eta_{G}^{2}$ = 0.0007, seems to indicate that for low similarity categories interleaved performance is better for old items in the immediate transfer test when compared to blocked study for the same type of items but this difference disappears for the 24-h delayed test session.

Finally, performance is overall better for old stimuli compared to new ones, *F*(1,111) = 135.90, *p* < 0.0001, $\eta_{G}^{2}$ = 0.04. This result is indicative that, at least in part, participants may be memorizing individual exemplars during study. Interestingly, the difference in performance between new and old stimuli is greater for low similarity categories compared to high similarity categories, *F*(1,111) = 75.87, *p* < 0.0001, $\eta_{G}^{2}$ = 0.02. These results suggest that, given the greater number of discrimination points between individual stimuli in the low similarity categories, participants are more likely to have better differentiated individual memories for the low similarity stimuli.

Overall the results from this experiment show an interaction between the schedule of study and the type of category on test performance, which remains unaltered with increases in retention interval. Moreover, there is no overall increase in the benefits of interleaved study with increased retention intervals. Performance during the transfer tests also does not seem to be the result of the differential difficulties found during study. There is an interaction between type of category and schedule of study at test, which is not seen during study.

## EXPERIMENT 1B

We designed Experiment 1B to investigate the possibility that the findings in Experiment 1A for the delayed tests are in part the result of the existence of a Refresher section immediately before those tests and not the learning that took place in the previous day. In this experiment a new group of participants completed only the second day session of Experiment 1A. If the Refresher presented during this session were sufficient for participants to learn the categories, then we should see similar results here to what was found for the delayed test of Experiment 1A. On the contrary, if the brief refresher section is not enough for participants to effectively learn the categories, we would expect a qualitative decline in performance compared to Experiment 1A, as well as no performance differences between the two schedules of study and no interaction between schedule of study and category type during test, contrary to what is seen for Experiment 1A.

### METHOD

#### Participants

A total of 63 Indiana University undergraduate students, who had not participated in the previous experiment, volunteered to participate in this study in exchange for partial course credit. Participants were randomly assigned to either the low similarity (*N* = 26) or high similarity (*N* = 37) conditions. No exclusion criteria were used to match inclusion criteria in the second day of Experiment 1A.

#### Apparatus and stimuli

The same set of stimuli as in Experiment 1A were used in this experiment.

#### Design and procedure

This experiment had two conditions manipulated within-participants (interleaved vs. blocked study), and two condition manipulated between-participants (high similarity vs. low similarity categories). Participants completed a task similar to the second session of Experiment 1A. Participants started by completing a short study task (the refresher task in Experiment 1A) for three of the categories followed by immediate test for those categories and then repeated these steps for the second group of three categories. Half the participants started with interleaved study of the categories and the other half with blocked study of the categories. All other details not presented here were the same as in Experiment 1A.

### RESULTS AND DISCUSSION

**FIGURE 4:**
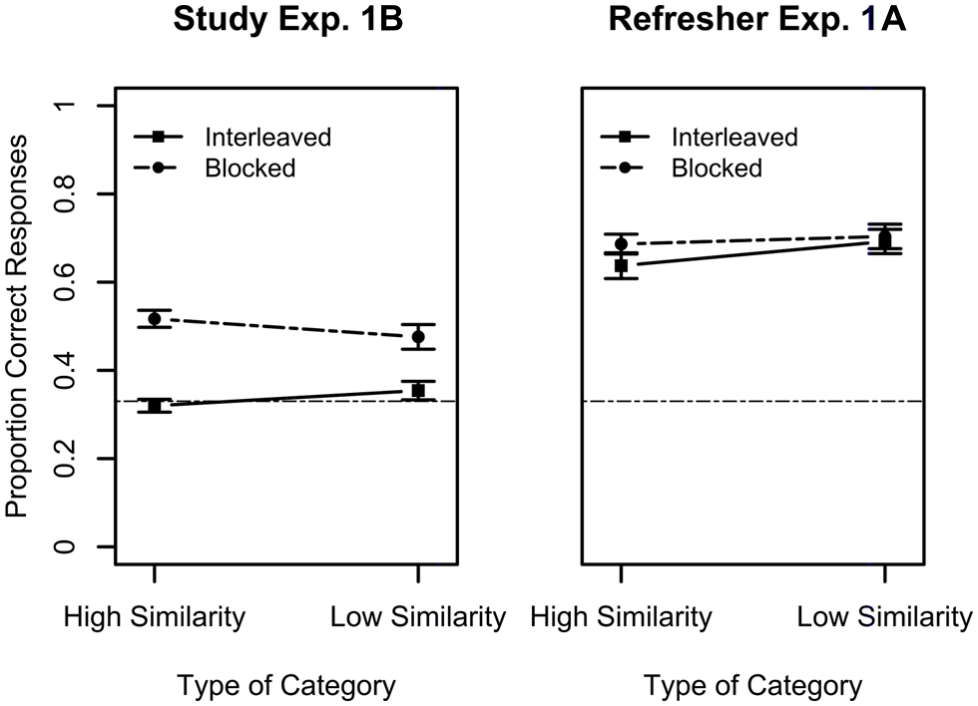
**Performance during the Study Phase of Experiment 1B for high and low similarity categories (left panel).** The right panel presents data from the second day refresher only presented in **Figure 2** and is depicted here for comparison purposes. Solid lines indicate interleaved study, while dashed lines indicate blocked study. Error bars indicate standard errors of the means. Chance-level performance in this task was 0.33 and is represented in the graphs by the horizontal dashed line.

The results for the study phase of Experiment 1B are depicted in **Figure 4** (in which the results of the refresher phase of Experiment 1A are also depicted for comparison). As it can be seen from the **Figure 4**, performance is qualitatively worse during study in Experiment 1B compared to performance in the refresher phase of Experiment 1A. Moreover, performance in Experiment 1B is overall better during blocked study when compared to interleaved study, *F*(1,61) = 58.84, *p* < 0.0001, $\eta_{G}^{2}$ = 0.33. The main effect of category structure and the interaction between the two variables were not reliable (both *p*s > 0.05). We also compared performance with chance level of 33% for each condition and type of category combination. Performance was reliably above chance only in the case of the blocked study condition, *t*(25) = 5.22, *p* < 0.0001 for low similarity categories and *t*(36) = 9.66, *p* < 0.0001 for high similarity conditions.

**FIGURE 5:**
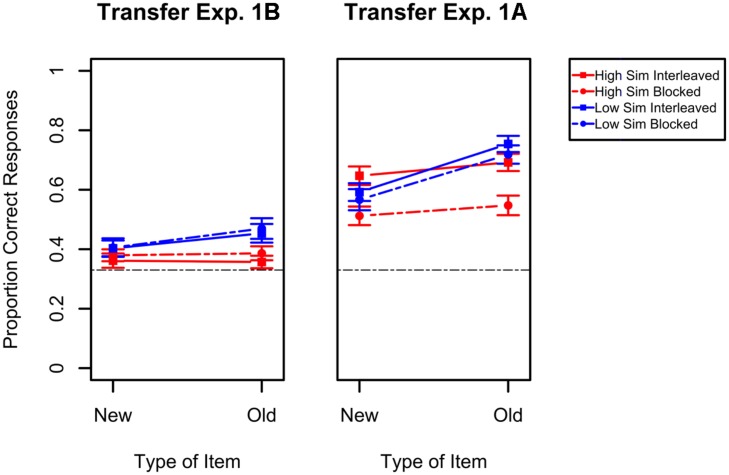
**Performance in the transfer task of Experiment 1B (left panel).** The right panel depicts results for the delayed transfer of Experiment 1A for comparison purposes. Results for the high similarity categories are presented in red while results for the low similarity categories are presented in blue. For each of these, the dashed lines represent blocked study while the solid lines represent interleaved study. Error bars indicate standard errors of the means. Chance-level performance in this task was 0.33 and is represented in the graphs by the black dashed line.

Turning now to performance during the immediate transfer test, the results indicate that overall participants’ performance following only the refresher task is considerably worse than in the delayed test of Experiment 1A and close to chance. The results of the transfer task are presented in the left panel of **Figure 5** along with the results from the delayed transfer test of Experiment 1A (right panel) for comparison. A mixed ANOVA with type of item (new vs. old) and study schedule (interleaved vs. blocked) as within-subject factors and category structure as a between-subject factor for the results of Experiment 1B only showed an effect of category structure, *F*(1,61) = 6.67, *p* = 0.01, $\eta_{G}^{2}$ = 0.04, with better performance for low similarity categories, and type of stimuli, *F*(1,61) = 5.55, *p* = 0.02, $\eta_{G}^{2}$ = 0.01, with better performance for old items. Moreover, the interaction between these two variables was also reliable. It was only when items were both old and had low similarity that categorization accuracy was appreciably above chance. When only one of these factor levels was present, accuracy was close to chance, *F*(1,61) = 5.15, *p* = 0.03, $\eta_{G}^{2}$ = 0.009. No other main effect or interaction was statistically reliable (all *F*s < 0). Overall performance is only slightly above chance, considerably worse than what is seen in the second day of Experiment 1A, and no effect of study schedule or category structure were found. This demonstrates that the results found for the delayed transfer test of Experiment 1A are unlikely to be the result of the short refresher study session but rather are the result of the extensive learning phase that took place 24 h earlier.

## GENERAL DISCUSSION

Taken together, the results presented here suggest that (a) the advantage of increased temporal lag between repetitions of the same category is not being masked by the use of an immediate test with low similarity categories – there was no difference between immediate generalization and a 24-h delayed generalization for any of the category structures. Similarly, (b) there was no overall increase in interleaved study benefits with an increase in retention interval, unlike previous evidence with verbatim repetitions. In addition, (c) different study sequences change the relative emphasis on different properties of the category items as seen by the relative learning benefit of each schedule, measured by generalization to novel items.

As we mentioned in the Section “Introduction,” in the context of verbatim repetitions, greater temporal delays between repetitions improves memory, particularly when the relative difference between the temporal lag during study and the temporal lag between study and test is increased (e.g., Crowder, 1976; Glenberg, 1976; Murray, 1983). However, in the current experiments we did not see such an effect of increased retention interval, which questions the importance of temporal spacing during study for the benefits of interleaved study in category learning. This finding is in agreement with recent results by Kang and Pashler (2012), and Birnbaum et al. (2013) showing that introducing an additional temporal delay between presentations during interleaved study results in memory performance similar to that of a blocked study condition (i.e., decreases the interleaved advantage). One possibility is that 24 h is too short and with longer retention intervals an advantage of longer temporal spacing between categories would be seen. Though this remains an open question for future research and the exact forgetting function for this type of stimuli is unknown, we believe that it is unlikely that longer delays would yield interleaved study benefits since 24 h has been demonstrated to be sufficient before (Ste-Marie et al., 2004) and previous studies show noticeable increases in the benefits of short temporal spacing during study with 24-h retention intervals (see Cepeda et al., 2008).

However, even though temporal spacing by itself might not play a fundamental role in the interleaved advantage seen thus far, the importance of the temporal delay between repetitions during study should not be ignored. For instance, Vlach et al. (2012) showed that introducing a temporal delay between different exemplars of the same category resulted in improved performance in a 15 min delayed generalization test. The authors taught 2 year-old children eight different categories organized around shape, each containing four similar exemplars varying in other properties (color, texture, and size). Different groups of children learned the categories either by studying all the exemplars simultaneously, individually blocked by category, or spaced (similar to the blocked condition but a play time was introduced after each naming trial). Children were tested (1) immediately after learning each category (i.e., after learning the first category a test session for that category would take place, prior to teaching the next category), and (2) 15 min later. For immediate tests, simultaneous presentation resulted in better generalization performance. Interestingly, 15 min later, only children in the spaced condition were able to generalize the categories learned above chance level. In fact, performance in the spaced condition did not seem to diminish from the first to second test, while it decreased considerably for both blocked and simultaneous presentations.

Birnbaum et al. (2013) found similar results with college students using natural categories. In one experiment the authors contrasted blocked and interleaved study when implemented contiguously with another condition in which a temporal delay was introduced between repetitions either of the same category (blocked + spaced) or different categories (interleaved + spaced). While interleaved + spaced resulted in worse performance than interleaved (for similar results see Kang and Pashler, 2012), the opposite pattern was seen for the blocked study conditions, i.e., blocked + spaced resulted in better performance than blocked study. This evidence across development and stimuli makes it apparent that forgetting and retrieval of information during study might play a role in learning differences seen with different sequencing schedules during study. As we mentioned in the Section “Introduction,” participants might engage in a process of interactive recall in which features of the previous encounter with that category are recalled when a new item of the same category is presented.

Overall, the present results are in agreement with the attentional bias hypothesis proposed by Carvalho and Goldstone (2014a,b) that predicts that the benefits of interleaved vs. blocked study are the result of an attentional biasing process taking place during the study phase. The attentional bias hypothesis proposes that during inductive category learning, learners tend to establish relations between the current example being studied and the previous one. If the two objects belong to the same category, the learner’s attention will be focused on similarities. If, conversely, the two stimuli belong to different categories, the learner’s attention will be focused on the differences between the two objects. In this way, *across time*, attention will be increasingly biased towards relevant within-category similarities and between-category differences. This will affect category representation, which will, in turn, affect category encoding and recollection. With each new trial, categorization relevant properties will be progressively better encoded while irrelevant ones will be poorly or not encoded at all. Thus, blocked study emphasizes mostly similarities within categories, benefiting the acquisition of low similarity categories, while learning high similarity categories will be improved by attending to differences between categories during interleaved study.

In this work we used novel, lab generated, category stimuli presented briefly on the screen. While this type of stimulus and procedure matches current research in the concept learning literature, it may limit generalization. It is possible that using natural categories not defined by a rule, in which the stimuli are presented for a longer period of time or participants do not have to guess the category assignment during study, might provide different results (but see, Carvalho and Goldstone, 2011, 2014b; Kang and Pashler, 2012; Birnbaum et al., 2013; Rawson et al., 2014). Moreover, it is possible that the Refresher included before the 24 h-delayed test interacted with the type of study during the initial training session influencing the results seen for the delayed test. While this hypothesis cannot be ruled out by the present results, given that the same schedule of study was used during the Refresher as during the initial study session and the Refresher by itself did not allow participants to learn the category structures (Experiment 1B), we believe the possible influence of the Refresher is minimized. In addition, the inclusion of a Refresher might present added educational validity to the results presented here. Students often review the concepts immediately before the examination, regardless of when the initial study took place.

As a theoretical framework, one possible way to integrate the benefits of temporal spacing and the benefits of sequential comparisons is by hypothesizing that they result from different learning processes, happening simultaneously during category acquisition. Successfully learning new categories can be achieved by encoding the relevant features and rules or by encoding individual exemplars that will be compared to novel instances for novel categorizations in the future. Within exemplar models of category learning, both of these alternatives would depend on whether one feature (or set of features) was selectively attended during study, or all features were equally weighted (Nosofsky et al., 1989; Nosofsky, 1991; Kruschke, 1992).

At a first pass, learners might try to identify and isolate the relevant properties of the stimuli for categorization. The relevant properties are the similarities within categories for low-similarity categories and differences between categories for high-similarity categories. Identifying these properties will be promoted by specific sequential comparisons as discussed before. If learners are successful, these relevant parts will receive greater attentional resources and be more efficiently encoded. Participants can then look for those when categorizing novel stimuli during a subsequent transfer task. However, under some situations (blocked study of high similarity categories and interleaved study of low similarity categories), the relevant properties do not receive as much attention. This might lead participants to encode more features of each individual exemplar – a prediction derived from exemplar models assuming equally distributed attentional weights to all the features. This encoding would be improved by adding temporal spacing between presentations, which will result in increased effort in retrieving previous encounters during the recursive retrieval process and thus a better encoding of each stimulus (Bjork and Allen, 1970; Cuddy and Jacoby, 1982; Krug et al., 1990). These exemplar memories of each stimulus can then be used to categorize new stimuli during transfer.

Coherent with this proposal, in Experiment 1A as well as in previous work (Carvalho and Goldstone, 2014a), when low similarity categories where used, memory for old items was best following interleaved study than blocked study. This, although not definitive, is indicative that, when abstracting the relevant feature during study is not possible, learners might encode the entire stimulus, benefiting from manipulations that increase memory for individual stimuli. Perhaps a critical difference between these two processes is whether category abstraction is possible during study, which allows for encoding only the relevant features, or takes place only during test. This might be analogous to the results demonstrating differential exemplar memory for items that fit an abstracted categorization rule and those which do not (Palmeri and Nosofsky, 1995; Blair and Homa, 2003; Sakamoto and Love, 2004).

An important venue for future work would be to systematically contrast memory and generalization for different category structures by increasing and decreasing temporal spacing between successive presentations. One prediction deriving from the proposal presented here would be that memory for the relevant feature encoded during study would be better for blocked study of low similarity categories and interleaved study of high similarity categories. Conversely, memory for the whole exemplars would be better for interleaved study of low similarity categories and blocked study of high similarity categories. Additionally, increasing the temporal spacing would have a positive effect for individual memories of each stimuli studied while a negative effect on memory for the abstracted category-relevant feature.

Information is usually presented to us in a structured, ordered, way and it is likely that this order will shape how and how well we learn. In inductive category learning, the sequence of category examples has the potential to change what is encoded (Elio and Anderson, 1984; Medin and Bettger, 1994). Different schedules promote different attentional biases due to different sequential ordering, and change how information is encoded and remembered due to different temporal spacing between category repetitions. The results presented here show that increasing the temporal delay between study and test does not change the differential benefits of interleaved over blocked study for different types of categories. However, we propose that even though these results are consistent with the idea that the spacing effect does not play a role in the interleaved advantage for our task, retrieval and forgetting during study are likely to play a role in study sequencing effects in category learning. We presented a conceptual framework that integrates the effects of temporal spacing between repetitions during study as well as exemplar contrast.

## Conflict of Interest Statement

The authors declare that the research was conducted in the absence of any commercial or financial relationships that could be construed as a potential conflict of interest.

## Footnotes

1 We opted to include a refresher to remind participants of the mapping between blob groupings and category labels because we are interested in how well participants learned the groupings, i.e., category structure, and not whether the mapping between the learned structure and the category label is also maintained. Memorizing novel names when learning groupings of novel stimuli is a demanding task (Ashby and O’Brien, 2005) and learners’ ability to categorize new items is more influenced by changes in how the objects are grouped than the labels used (i.e., changing the labels for group A and B is less detrimental than mixing the items from group A and B into new categories, e.g., Hendrickson et al., 2012).
